# The motion after-effect: local and global contributions to contrast sensitivity

**DOI:** 10.1098/rspb.2008.1932

**Published:** 2009-02-25

**Authors:** Karin Nordström, David C. O'Carroll

**Affiliations:** Discipline of Physiology, School of Molecular and Biomedical Science, The University of AdelaideAdelaide, South Australia 5005, Australia

**Keywords:** spikelets, spike generation, after-potential, contrast threshold, adaptation, waterfall effect

## Abstract

Motion adaptation is a widespread phenomenon analogous to peripheral sensory adaptation, presumed to play a role in matching responses to prevailing current stimulus parameters and thus to maximize efficiency of motion coding. While several components of motion adaptation (contrast gain reduction, output range reduction and motion after-effect) have been described, previous work is inconclusive as to whether these are separable phenomena and whether they are locally generated. We used intracellular recordings from single horizontal system neurons in the fly to test the effect of local adaptation on the full contrast-response function for stimuli at an unadapted location. We show that contrast gain and output range reductions are primarily local phenomena and are probably associated with spatially distinct synaptic changes, while the antagonistic after-potential operates globally by transferring to previously unadapted locations. Using noise analysis and signal processing techniques to remove ‘spikelets’, we also characterize a previously undescribed alternating current component of adaptation that can explain several phenomena observed in earlier studies.

## 1. Introduction

Sensory neurons and systems adapt to prolonged stimulation. Such dependence on stimulus history matches neural sensitivity and response properties to the strength and statistics of current stimulus parameters. While it has been argued that adaptation operates on different time scales to make information processing efficient (e.g. de Ruyter van Steveninck *et al.* 1986; Brenner *et al.* 2000), recent studies challenge this view and show that many phenomena observed during stimulation can be explained by the nonlinearity inherent to motion detection (Borst *et al.* 2005; Safran *et al.* 2007). However, there is little doubt that motion adaptation gives rise to several distinct effects on subsequent responses to stimuli that are less easily explained, including a variety of motion after-effects (MAEs) such as the famous ‘waterfall effect’ in human psychophysics (for a review, see Mather *et al.* 1998). These after-effects following prolonged exposure to motion have been studied extensively in a range of animals (e.g. rabbit: Barlow & Hill 1963; macaque: Kohn & Movshon 2003; fly: Maddess & Laughlin 1985). Earlier electrophysiological studies of transient antagonistic after-responses, characteristic of direction-selective visual neurons, suggested that these could explain the waterfall effect (Barlow & Hill 1963). However, the specific mechanisms and location of motion adaptation remain poorly studied. In particular, while earlier studies revealed clear evidence that some components of adaptation must be locally generated (Maddess & Laughlin 1985), more recent work (e.g. Harris *et al.* 2000; Reisenman *et al.* 2003; Borst *et al.* 2005; Neri & Laughlin 2005; Kurtz 2007; Kalb *et al.* 2008) has unravelled several distinct mechanisms contributing to adaptation and the degree to which these are locally or globally generated remains unclear.

The fly visual system provides a superb physiological model for studying adaptation. Importantly, most data obtained to date support similar mechanisms operating in motion analysis and adaptation by both mammalian and insect visual systems (Clifford & Langley 1996; Clifford & Ibbotson 2002). In vertebrate cortex recordings, response profiles of individual cells vary to a large extent (e.g. compare individual response functions with the same stimuli in Kohn & Movshon 2003), thus requiring population-level analysis to draw reliable conclusions. Fly direction-selective lobula plate tangential cells (LPTCs) detect wide-field motion by spatially pooling across a large part of the visual field. As they are readily identified based on their physiological response properties, the data can be pooled across recordings from the same neuron in different individuals. Furthermore, several classes of LPTCs forming the horizontal system (HS) and vertical system (VS) are large enough for reliable intracellular recordings *in vivo*, and respond with large direction-opponent graded responses (e.g. Hausen 1982; Hengstenberg 1982; Egelhaaf & Kern 2002), allowing detailed investigation of phenomena below the threshold for action potential generation (see, e.g. Warzecha & Egelhaaf 2001).

**Figure 1:**
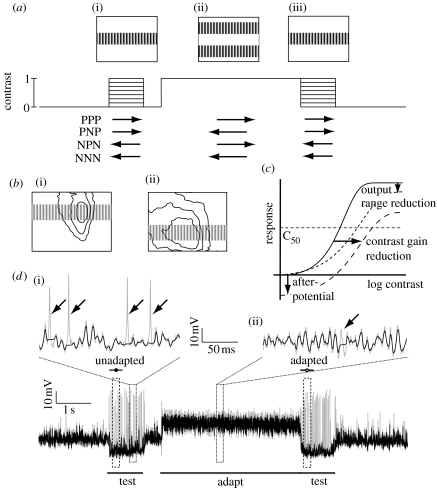
Protocol for testing the local effects of motion adaptation. (*a*) Our test-adapt-test protocol uses an adapting sine-wave grating (0.1 cpd, 20 Hz, contrast=1.0) with a ‘notch’ in the middle, thus leaving a large part of the neuron unadapted. The test stimulus consists of a sine-wave ‘strip’ (0.1 cpd, 5 Hz) covering this unadapted part. We vary the test contrast in eight steps between 0 and 1.0. The testing and adapting stimuli are displayed in four combinations of preferred (P) and anti-preferred (null, N) directions of motion ((i, iii) test, 5 Hz; (ii) adapt, 20 Hz). (*b*) The test stimulus is placed over the centre of the receptive field of male (i) HSN and (ii) HSNE. The contour lines show 25, 50 and 100% sensitivity amplitude of averaged receptive fields (Nordström *et al.* 2008). (*c*) Diagram of the three motion adaptation components described by Harris *et al.* (2000), with the unadapted (solid curve), adapted (long-dashed curve) and normalized (short-dashed curve) responses shown. The after-potential shifts the curve vertically, while the contrast gain reduction generates a horizontal shift, and the output range reduction compresses the gain. (*d*) The data trace (grey) shows the intracellular response of a male HSN to the NPN protocol. The unadapted and adapted test response times are delineated (100–300 ms). For most analyses we use the graded membrane potential after removing spikelets (black trace). The two magnifications show the efficiency of such spikelet removal during (i) anti-preferred motion and (ii) preferred direction motion (arrows point to successfully removed spikelets).

In physiological terms, visual motion adaptation can be broken down into several components (figure 1*c*; see fig. 3 in Harris *et al.* 2000): (i) contrast gain reduction, which follows adaptation in any direction; (ii) an antagonistic (i.e. direction-selective) after-potential (analogous to the waterfall effect observed in human psychophysics); and (iii) an output range reduction. Adaptation may operate at several levels of the motion detection pathway both through mechanisms located pre-synaptic to motion-sensitive neurons, but potentially also within the neurons themselves (e.g. Harris *et al.* 2000; Kohn & Movshon 2003; Reisenman *et al.* 2003; Kurtz 2007). As contrast gain reduction follows adaptation in either direction it has been suggested to originate at stages prior to the computation of motion direction, i.e. upstream of fly LPTCs (Harris *et al.* 2000). In vertebrates, contrast gain reduction has been described in the magnocellular (M) pathway (Solomon *et al.* 2004) and in the middle temporal (MT) area, at least following preferred direction stimulation (Kohn & Movshon 2003). The second component, the antagonistic after-potential, is larger following preferred than anti-preferred direction adaptation (Harris *et al.* 2000; Kohn & Movshon 2003) and either originates in the motion neurons themselves (Kurtz 2007) or at earlier processing stages. In vertebrates, the MAE is found already in the M pathway (Solomon *et al.* 2004) and psychophysics suggests this to be a global phenomenon (Smith *et al.* 2000). The origin of the third component of motion adaptation, output range reduction, is unknown (Harris *et al.* 2000).

Although earlier studies suggested that adaptation is a local phenomenon (Maddess & Laughlin 1985; Reisenman *et al.* 2003), a recent study showed that local adaptation exerted global influences on the directional gain of responses in previously unstimulated parts of the receptive field (Neri & Laughlin 2005). Another recent study testing the effects of adaptation at the same location (Kalb *et al.* 2008) described similar changes in directional sensitivity that can accounted for by the motion adaptation components identified by Harris *et al.* (2000). However, both studies (Neri & Laughlin 2005; Kalb *et al.* 2008) examine the effects using high-contrast patterns and extracellularly recorded responses of spiking LPTCs (H1 and V1), making it difficult to distinguish between the effects due to changes in gain (either local or global) and nonlinear behaviour of the spike-generating mechanism (see discussion in Kretzberg *et al.* 2001). The graded responses of HS and VS cells provide an ideal model for analysis of the underlying generator potential and response components below the threshold for spike generation, particularly in the anti-preferred direction where high-contrast stimuli typically invoke complete suppression of spiking responses in LPTCs (Hausen & Egelhaaf 1989).

In this paper, we examine the effect of local adaptation on the full contrast-response function with intracellular recordings of fly HS neurons. We define local phenomena as those components of motion adaptation that remain spatially separated within the receptive field, while global components transfer to previously unstimulated locations. Testing the effect of adaptation on the full contrast sensitivity function has been shown to be a powerful tool for isolating the different components of motion adaptation (Harris *et al.* 2000). Here, we show that contrast gain and output range reductions are primarily local phenomena and probably associated with spatially distinct synaptic changes. Using noise analysis and signal processing techniques to remove ‘spikelets’, we also characterize a previously undescribed alternating current (AC) component of adaptation that can explain some of the phenomena observed by others. This AC component and the antagonistic after-potential act globally in the neuron by transferring to previously unstimulated locations.

## 2. Material and methods

### (a) Electrophysiology

Male hoverflies (*Eristalis tenax*) were collected under permit from the wild (the Botanic Gardens of Adelaide) and kept in the dark at 4°C until experimental time. The animals were waxed down with the head tilted forward and a small hole was cut over the left lobula complex leaving the neural sheath intact. Horizontal system north (HSN) and horizontal system north equatorial (HSNE) neurons (Nordström *et al.* 2008) were recorded intracellularly from the axon or main dendrites using aluminium silicate micropipettes pulled on Sutter Instruments P-97 and filled with 2 M KCl. Electrodes had a typical tip resistance of 120 MΩ and were inserted with a Piezo micromanipulator. Flies were mounted 14 cm in front of an RGB CRT display with a mean luminance of 90 Cd m^−2^. Visual stimuli were generated using the public domain software package VisionEgg (www.visionegg.org). The monitor subtended 100×75° at the fly's central visual field, with a resolution of 640×480 pixels and a refresh rate of 200 Hz. The data were digitized at 5 kHz using a 16 bit A/D converter (National Instruments) and analysed off-line with Matlab.

### (b) Visual stimuli

We used test-adapt-test protocols based on those used by Harris *et al.* (2000) to determine the effect of adaptation on HS neurons (figure 1*a*). The 1 s test stimulus consisted of a drifting sine grating matching the spatio-temporal optimum for fly HS cells (0.1 cpd, 5 Hz), covering the entire width of the monitor and with a vertical extent of 24°. We varied the contrast of the test stimuli in eight intervals logarithmically spaced between contrasts of 0 and 1.0. This test grating was positioned vertically at the centre of the receptive field determined at the start of each experiment. Because the receptive fields of the HSN and HSNE neurons span a large part of the vertical extent of the display (Nordström *et al.* 2008), this test stimulus is thus confined to approximately one-third of the receptive field in both cell types (figure 1*b*). The adapting stimulus consisted of a high-contrast (*C*=1.0), high-velocity sine grating (0.1 cpd, 20 Hz), covering the remaining receptive field above and below the test region (i.e. with a height of 72°, but excluding the middle strip of 24°).

We used four different conditions in which the first and second test stimuli were identical and either in the preferred (P) or anti-preferred/null (N) directions, with the test being either the same or opposite direction to the adaptor. We refer to these conditions using abbreviations based on the test–adapt–test directions, hence: null test–null adapt–null test (NNN); null test–preferred adapt–null test (NPN); preferred test–null adapt–preferred test (PNP); and preferred test–preferred adapt–preferred test (PPP) (figure 1*a*). Between the trials, the monitor was left at mid-luminance (90 Cd m^−2^) for a minimum of 6 s to allow the neuron to recover from adaptation.

As controls, we did some experiments in which we tested local adaptation by using the same combinations of stimuli, but where both test and adapting stimuli were limited to the central ‘strip’. Since the zero contrast test condition in the above experiments generates powerful local flicker stimuli at the onset and offset of the adapting pattern, we also tested the effect of a stationary test pattern at maximum contrast (a condition more akin to those in which the MAE is typically observed psychophysically).

### (c) Data analysis

We only kept data from (i) the male HSN and HSNE (these neurons showed no differences in response properties), (ii) neurons that gave a minimum 10 mV response to an optimal stimulus, (iii) neurons where all four test conditions were completed (this enabled paired *t*-tests), and (iv) neurons where we recorded no drift of resting membrane potential, or reduction in response to control stimuli.

HS neurons display large graded membrane potential changes upon which variable degrees of ‘spikelet’ events ride. The origin and functional significance of spikelets remains a topic for further investigation, but it is likely that they are associated with regenerative conductances in the axon of the neuron (Haag & Borst 1998). Since they are primarily monophasic depolarizing events, we attempted to separate the spikelet component from the underlying graded response (‘generator’ potential) by detecting and subtracting spike-like events in the raw data before averaging several repetitions of the same stimuli within each neuron (data trace in figure 1*d*). The spike detection algorithm (written in Matlab; Nordström *et al.* 2006) used an adaptive combination of level and edge detection mechanisms that allowed it to detect events of varying shape and amplitude. This method sets a 10 ms window to a noise-free flat line. However, the large variability of spikelet shape made it impossible to define parameters that detect and account for all transient depolarizing events, especially during strong preferred direction stimulation where such events were small (i.e. when the neuron is strongly depolarized; figure 1*d*). After filtering, we averaged the membrane potential between 100 and 300 ms after stimulus onset (Harris *et al.* 2000). In each neuron, we performed three to six repetitions of each stimulus, pooled to give one measurement. Given *n* numbers thus represent number of animals, not individual trials. We display all the data as mean±s.e.m. unless otherwise stated.

We determined unadapted and adapted responses to different contrasts with the unadapted response to a test contrast of 1.0 defined as the maximum response. We interpolated the data with a quadratic spline solved to determine C_50_, the contrast that gives 50 per cent maximum response. Contrast sensitivity was also solved at a ‘detectability’ criterion based on 1.5 times the standard deviation (1.5×s.d.) in the unstimulated membrane potential (i.e. when the neuron viewed a blank screen). To determine shifts in contrast sensitivity we performed paired *t*-tests followed by a *post hoc* Bonferroni correction with significant differences assigned to *p*<0.05.

### (d) Noise analysis

To investigate the contribution of spikelets to response power before, during and after motion in either direction, we performed fast Fourier transform (FFT) analysis of the raw data before and after spikelet removal. The peri-stimulus analysis was based on the last 3 s (out of 4 s) of the adapting time in experiments where the test contrast was 0. The MAE response power was measured between 0.1 and 1 s following the end of the adapting stimulus in the same experiments. To quantify the differences between the conditions, we measured the mean power spectral density between 95 and 105 Hz, with significance defined as *p*<0.05 (after Bonferroni correction).

## 3. Results and discussion

### (a) Testing local and global effects on adaptation

Our stimulus (figure 1*a*) enabled us to differentiate local and global effects of motion adaptation by testing and adapting in spatially distinct parts of the receptive field of individual HSN and HSNE neurons in male hoverflies. Although hoverflies differ from blowflies in their visual ecology, and recent work shows interesting sexual dimorphism of these neurons not found in *Calliphora* (Nordström *et al.* 2008), motion adaptation and underlying temporal coding is highly consistent across LPTCs and dipteran species. For example, Kalb *et al.* (2008) recently showed that the Harris components of adaptation revealed in *Eristalis* (Harris *et al.* 2000) are all observed in VS and V1 in *Calliphora*. Other studies suggest that motion adaptation in HS and H1 in *Eristalis* and *Calliphora* is comparable (Borst & Egelhaaf 1987; Harris & O'Carroll 2002) and even *Drosophila* LPTCs code motion similarly to larger flies (Joesch *et al.* 2008).

Although HSN and HSNE differ in receptive field shape and location (Nordström *et al.* 2008) we adjusted the vertical location of the stimulus so that the test was always centred within the receptive field (figure 1*b*). The adaptor grating (figure 1*a*) lacked the central strip, thus ensuring that the neuron was adapted in a different part of the receptive field, which we quantified online for each recorded neuron using the rapid method described by Nordström *et al.* (2008). Analysis of the data from the two neuron types revealed no significant differences, so we pooled all the data for subsequent analysis.

The data trace in figure 1*d* shows the response of a single male HSN neuron to an anti-preferred (null) test pattern and a preferred direction adaptor (the NPN condition in figure 1*a*). HS neurons give primarily graded responses, hyperpolarizing to anti-preferred and depolarizing to preferred direction motion. Resting membrane potential is unusually high (−53±1.9 mV, *n*=17) compared with typical spiking neurons in the same brain region. Small spike-like events (spikelets) ride upon the graded responses (Haag & Borst 1998). Unlike discrete post-synaptic potentials, which are not observed in axonal recordings from HS neurons, these spikelets are generated within the dendrites by voltage-gated sodium currents and are then amplified by the axon via additional voltage-gated currents (Haag & Borst 1996, 1998). As in this earlier work, we found that neurons with the lowest membrane potential and largest graded responses also produce more regular and larger amplitude action potentials when stimulated in the anti-preferred direction (i.e. when strongly hyperpolarized), in addition to irregular spikelets. Depolarizing stimuli evoke frequent, smaller and less regular spikelets (figure 1*d*).

The role of spikelets in neural coding by fly tangential neurons has been the subject of some discussion in recent years (e.g. Warzecha & Egelhaaf 2001). Haag & Borst (1996) proposed a role in amplifying depolarizing transient events, and showed that small irregular spikelets can be transformed into full-blown action potentials by hyperpolarizing the cell (Haag & Borst 1998). Since spikelets are probably generated post-synaptically (Haag & Borst 1996; Beckers *et al.* 2007), their highly variable nature (even in the recordings of the same neuron in different flies) complicates analysis of the underlying graded response. In an attempt to analyse the graded responses independent of the presence of spikelets, we applied a detection and filtering technique (see §2) that sets local membrane potential in the vicinity of the detected event to that immediately preceding it (i.e. the generator potential) before averaging the membrane potential within the desired peri-stimulus response period. The data trace in figure 1*d* shows the data before (grey) and after (black) such spikelet removal. While the large monophasic action potentials generated during hyperpolarized responses to anti-preferred direction motion are relatively easy to identify and remove (figure 1*d*, arrows), the smaller and irregularly shaped spikelets generated during either direction of motion are much harder, and we were unable to set filter parameters that are 100 per cent effective in removing their influence from the analysis of the underlying membrane potential.

### (b) Contrast gain reduction

To determine whether the three previously identified components of adaptation (i.e. non-directional contrast gain reduction, antagonistic after-potential and output range reduction; see figure 1*c* and Harris *et al.* 2000) are generated locally, we measure the contrast sensitivity functions for the four possible combinations of preferred (P) or anti-preferred (or null, N) test or adapting stimuli (PPP, PNP, NPN and NNN in figure 1*a*).

**Figure 2:**
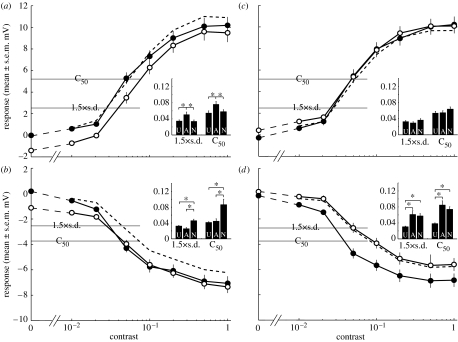
Local adaptation generates little contrast gain reduction. The graphs show the contrast sensitivity functions to the four test-adapt-test combinations: (*a*) PPP (preferred direction test and adapt), (*b*) NPN (anti-preferred direction test, preferred direction adapt), (*c*) PNP (preferred direction test, anti-preferred direction adapt) and (*d*) NNN (anti-preferred direction test and adapt) to the strip test and notch adapt stimuli. Unadapted (U; filled circles) refers to the response to the first test, and adapted (A; open circles) to the second test, after adaptation has taken place. The dashed lines (normalized, N) show the adapted response after subtracting the after-potential, i.e. the adapted response to a test contrast of zero. We use two measures to determine shifts in contrast sensitivity: C_50_ is calculated as the contrast that gives 50% maximum response (defined as the unadapted response to a test contrast of 1.0) and 1.5×s.d. is a ‘detectability’ criterion based on the standard deviation of the unstimulated membrane potential. The inset histograms show the contrast measured at C_50_ and 1.5×s.d. after fitting the data with a spline function. All data are displayed as mean±s.e.m., *n*=15. Asterisks indicate significant differences (*p*<0.05).

We quantify the first of these components, contrast gain reduction, by determining the contrast that evokes a 50 per cent maximal response (C_50_) and responses closer to absolute threshold, based on a ‘detectability’ criterion (1.5×s.d.; see §2). When the neuron is tested and adapted in the preferred direction, local adaptation shifts the contrast sensitivity function downwards and leads to a small but significant decrease in apparent sensitivity (figure 2*a*). However, a control stimulus where there is no test pattern (i.e. contrast of 0) reveals a hyperpolarizing after-potential of −1.4 mV. If we subtract this away from the adapted contrast sensitivity curve, the resultant line (figure 2*a*, dashed line) overlies the unadapted curve at low contrasts, with no significant difference in threshold. Hence, the small apparent reduction in contrast sensitivity is primarily due to the antagonistic after-potential, rather than a reduction in contrast gain. While this antagonistic after-potential may be generated locally, its effect is clearly global.

When we use the same adaptor, but test in the anti-preferred direction (figure 2*b*), responses reach a smaller maximum level (−7.1 mV, relative to the resting potential) of hyperpolarization (i.e. at contrast 1.0) compared with the degree of depolarization (+10.2 mV) observed for preferred direction stimulation. Since our filtering technique misses many smaller and less regular spikelets, particularly when the neuron is depolarized, and since such spikelets are monophasic depolarizing events (figure 1*d*, and see Haag & Borst 1996), they may boost the average magnitude of preferred direction responses and decrease those in the anti-preferred direction. Therefore, we need to be careful in comparing curves at supra-threshold response levels. Nevertheless, the post-adaptation response in this condition is consistent with the PPP condition: when there is no test stimulus, the adapted response is hyperpolarized compared with resting levels (−1.1 mV; figure 2*b*). From the curves it is evident that, as contrast increases, the additional hyperpolarization recruited by the test stimulus appears to shift absolute contrast thresholds to lower levels—apparently increasing sensitivity for low contrast stimuli. Again, however, if we subtract away the after-potential, this apparent increased sensitivity for lowest contrasts disappears (figure 2*b*, dashed line). As contrast increases further, the curves overlap. There is no significant difference in C_50_ or detectability between unadapted and adapted responses.

If we now adapt the neuron in the anti-preferred (null) direction (figure 2*c*,*d*) we observe much smaller shifts (0.3 mV) in the post-adaptation membrane potential, consistent with Harris *et al.*'s (2000) observation that the after-potential component of adaptation is direction-selective. In the preferred direction test condition (PNP; figure 2*c*), the adapted responses overlie the unadapted response over much of the response range. A very small depolarizing offset shows up at the lowest contrasts, but there is no significant difference in contrast sensitivity between unadapted and adapted responses. Interestingly, a larger and significant decrease in contrast sensitivity is evident when neurons are tested in the anti-preferred direction (figure 2*d*). Thresholds are significantly reduced whether or not we subtract away the small after-potential.

Since our spike filtering method is less effective at removing the spikelets for preferred direction stimuli, the NPN and NNN (figure 2*b*,*d*) conditions may provide a more reliable indicator of the sensitivity change following local adaptation. In both the cases, there appears to be a small decrease in contrast sensitivity, at least if we account for the contribution of the direction-selective after-potential (dashed lines, figure 2*b*,*d*). This shift is, however, much smaller than that observed by Harris *et al.* (2000) when test and adaptor stimuli are presented at the same location. We confirmed this to be the case for local stimulation in a subset of our recordings (*n*=4) by using the test strip stimulus to adapt the same part of the receptive field as that tested (see figure 1*a*,*b* in the electronic supplementary material). This revealed higher contrast sensitivity in the unadapted neurons than observed in the data from female flies by Harris *et al.* (2000), as expected given the ‘bright zone’ in the larger male eyes (Straw *et al.* 2006). Adapted responses, however, reveal a similarly large contrast gain reduction to that observed by Harris *et al.* (2000) and Kohn & Movshon (2003), confirming that locally induced gain reduction is not a sex-specific phenomenon. Furthermore, repetition of the spatially distinct adapt and test stimuli for a set of recordings from HS neurons in female flies elicit responses consistent with the male data in figure 2 (*n*=6; see fig. 1*c*,*d* in the electronic supplementary material).

While these findings confirm that contrast gain reduction is predominantly generated locally, the small reduction in contrast sensitivity evident from anti-preferred direction test data (figure 2*b*,*d*) suggests a possible global influence of local stimulation. Although our test and adapting stimuli are spatially distinct, local motion detectors at the inner boundaries of the adaptor might also be stimulated by the upper and lower edges of the test stimulus, perhaps explaining this small sensitivity reduction. To preclude this possibility, we repeated our experiment (*n*=4; see fig. 1*e*,*f* in the electronic supplementary material) with a more localized strip stimulus and a larger gap between the adapting grating such that stimuli were separated by 9°—larger than nearest or next-nearest neighbour interactions within the compound eye, where inter-receptor angles are approximately 1.1° (Straw *et al.* 2006). This dataset also shows a small contrast sensitivity reduction after normalizing for the after-potential (see fig. 1*f* inset in the electronic supplementary material), confirming that this component is expressed globally.

### (c) Antagonistic after-potential

**Figure 3:**
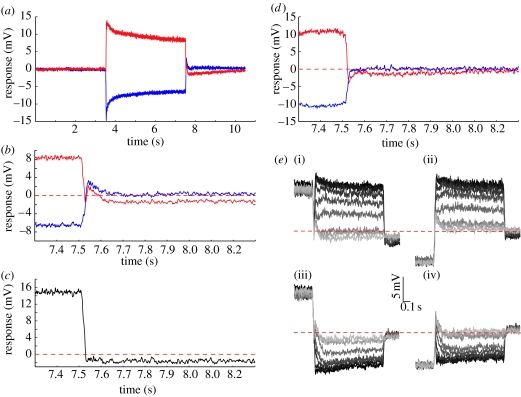
The antagonistic after-potential. (*a*) Average responses to adaptation (‘notch’ stimulus) in the preferred (red curve) and anti-preferred (blue curve) direction when the test contrast is zero. (*b*) A magnification of the responses surrounding the offset of adaptation (at 7.5 s) shows the asymmetry of the transient. (*c*) Responses to anti-preferred adaptation subtracted from those to preferred adaptation, and magnified around the offset of adaptation (at 7.5 s). (*d*) Average responses when adaptation is followed by a stationary test (0.1 cpd, contrast=1), magnified around the offset of adaptation (at 7.5 s). (*e*) Adapted responses following preferred adaptation ((i) PPP and (iii) NPN) and anti-preferred adaptation ((ii) PNP and (iv) NNN). The traces are shaded to illustrate the eight contrasts used (contrast of 0=lightest grey, to a contrast of 1.0=black). In (*a*–*e*), responses are averaged across 15 neurons, except in (*d*) where *n*=3.

Our data (figure 2) reveal subtle differences between preferred and anti-preferred direction sensitivity and confirm the direction-selective nature of the antagonistic MAE. This direction-selective component has been suggested to be generated within the LPTCs themselves, since input resistance is only decreased following stimulation in the preferred direction (Kurtz 2007). To investigate the potential role of this component further, we carefully examined the responses immediately following adaptation. Figure 3*a* shows the responses to the adapting stimulus alone averaged across 15 neurons. Although peri-adaptation responses are symmetrical about the resting potential, post-adaptation responses are clearly asymmetric and include an interesting transient component. Close examination of this transient (figure 3*b*) reveals a depolarizing component for either direction of stimulation. Following anti-preferred direction stimuli, this depolarizing transient decays to resting potential levels after 150 ms. Following preferred direction stimuli, the transient is similar in shape and duration, but it is superimposed on a much longer lasting hyperpolarizing potential. If we subtract away the anti-preferred from the preferred direction response (figure 3*c*) this transient disappears completely, suggesting that it is a direction-insensitive component, while the direction-selective MAE is evident within 30 ms of the cessation of motion.

What generates the non-directional transient? One possibility is that the local luminance flicker generated as the high-contrast adapting stimulus is replaced by a mid-luminance screen in these experiments might interact with the imbalance in the underlying motion detector mechanisms (Egelhaaf & Borst 1989) to generate transient responses. To test this hypothesis, we altered our stimulus so that the adapting pattern merely stopped but stayed visible at the end of the adapting period. Close inspection of the response shows that the depolarizing transient has disappeared (figure 3*d*). In this configuration, the hyperpolarizing after-potential following preferred direction adaptation resembles the difference signal described above (figure 3*c*). We thus conclude that the non-directional depolarizing transient is a flicker effect.

In contrast sensitivity experiments, this transient interacts with the response to the test stimulus in a complex way (figure 3*e*). As contrast increases, the transient is increasingly suppressed for all four conditions tested. Since the transient is consistently depolarizing (figure 3*b*), at low contrasts it contributes to the gain of preferred direction test stimuli, while decreasing the gain of anti-preferred direction stimuli (figure 3*e*). Since our analysis window was between 100 and 300 ms, the latter part of this transient is probably responsible for the small offset noted earlier at low contrasts following anti-preferred adaptation (figure 2*c*,*d*). Similarly, the stronger MAE might actually be underestimated following preferred direction adaptation (figure 2*a*,*b*).

### (d) Output range reduction

The origin of the third component of motion adaptation, output range reduction (figure 1*c*), remains unknown. Studies in the vertebrate M pathway show no output range reduction (Solomon *et al.* 2004), and in area MT only a slight response gain reduction (0.88) is found using spatially distinct PPP experiments similar to those described here (Kohn & Movshon 2003). In the fly, Harris *et al.* (2000) showed that the output range reduction following global adaptation is not associated directly with the contrast sensitivity reduction, since some stimuli (flicker and orthogonal motion) produce profound reductions in sensitivity without apparently reducing the saturation level of responses. They propose that it may instead result from the addition of an activity-dependent global conductance, which might be associated with the antagonistic after-potential. However, Kurtz (2007) recently showed that, while input resistance of HS cells is lower following stimulation with preferred direction motion, it is higher than resting levels following anti-preferred motion. If anything, this should boost the gain in PNP conditions, yet Harris *et al.* (2000) and Kalb *et al.* (2008) observed a reduction in output saturation to depolarizing test stimuli even following anti-preferred adaptation (fig. 2A in Harris *et al.* (2000); see fig. 1*a* in the electronic supplementary material). Interestingly, our equivalent local stimulus (figure 2*c*) shows no change in output range, with the adapted response perfectly overlying the unadapted. This argues very strongly against this component resulting from any global activity-dependent phenomenon within the HS neuron: it must be generated by local changes in either the local pre-synaptic inputs or within the dendrites of the neuron (see also discussion in Reisenman *et al.* 2003). Since we know that, this does not recruit a reduction in membrane resistance (Kurtz 2007), if these responses are the result of changes in the post-synaptic response, they must be due to a decrease in synaptic efficacy (e.g. local habituation of synapses).

### (e) A new AC component of adaptation

**Figure 4:**
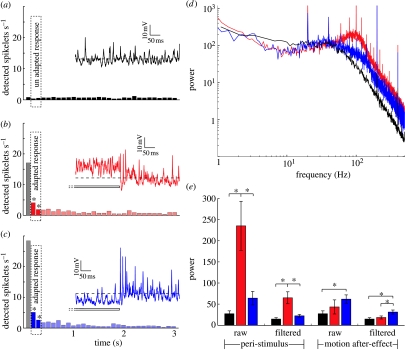
Spikelets shape the response profile during and after stimulation. (*a*) Average number of spikelets detected during 3 s in the unstimulated neuron. The spikelets are displayed in 100 ms bins (*n*=17). The inset shows the unadapted membrane potential of an individual HSN (same neuron as in figure 1*d*). (*b*) Average number of spikelets detected during 3 s following adaptation in the preferred direction. The first bin is greyed out owing to the presence of the non-directional depolarizing transient with associated spikelets described in figure 3. The spikelets are displayed in 100 ms bins, with bins from the 100–300 ms time period highlighted. Asterisks indicate significant difference compared with the unadapted neuron for these two bins (*p*<0.001, *n*=17). Inset shows 0.5 s of the response surrounding preferred direction motion offset in the same HSN. (*c*) Average number of spikelets detected during 3 s following adaptation in the anti-preferred direction. Spikelets are displayed in 100 ms bins, with bins from the 100–300 ms time period highlighted. Asterisks indicate significant difference compared with the unadapted neuron (*p*<0.001, *n*=17). The inset shows 0.5 s of the response surrounding anti-preferred direction motion offset. (*d*) FFT of raw data shows the power spectral density before (black curve, control; no stimulus) and during preferred (red curve) and anti-preferred (blue curve) direction adaptation (when there was no test, *n*=9). (*e*) Averaged power at 95–105 Hz before (control, black bars), during (peri-stimulus) and after adaptation (red bars, preferred direction motion; blue bars, anti-preferred motion). In each case, we show the power of the raw and the filtered (spikelets removed) data. Asterisks indicate significant differences (*p*<0.05, *n*=9).

Although our preferred direction stimuli reveal little evidence for an effect of adaptation on output range/saturation level, there is a consistent reduction in the strength of the response in anti-preferred responses following anti-preferred adaptation, leading to the significant increase in contrast thresholds noted earlier (NNN, figure 2*d*). Similar changes are observed for normalized data (after subtracting the after-potential) following preferred direction adaptation (NPN, figure 2*b*). Could these responses reflect changes in the influence of spikelets resulting from prolonged stimulation? The data in figure 3 are averaged across numerous presentations in many neurons, which would average all but the best correlated spike-like events to an apparent steady, graded depolarized response level (owing to the monophasic nature of these events; see Haag & Borst 1998). To address this question, we therefore need to consider responses in single trials. Qualitative examination of typical responses following control adapting stimuli (i.e. no test stimulus; figure 4*a*–*c*) shows that the response immediately following adaptation depends on the adapting stimulus. Following the prolonged hyperpolarization induced by anti-preferred direction motion, responses show a ‘burst’ of spikelets (figure 4*c*) compared with either the unstimulated (pre-adaptation; figure 4*a*) response, or the response following preferred direction motion (figure 4*b*). If we look at post-stimulus time histograms of detected spikelets during the 100–300 ms time window, this reveals a significant increase compared with the control after both types of adaptation (the second and third bins highlighted in the histograms in figure 4*b*,*c*). Importantly, the elevated spikelet rate lasts for several seconds and certainly outlasts the non-directional transient described earlier (figure 3), so it must be a separate phenomenon.

Given the variable nature of spikelet shape and the resultant imperfection of our spike detection method, we further investigate this component using Fourier analysis of the membrane potential before, during and after stimulation, and before and after application of our spikelet filtering technique. The power spectral density during preferred direction motion shows a broad peak at approximately 100 Hz compared with the unstimulated neuron (compare red curve with black curve in figure 4*d*). This is consistent with the resonant peak at similar frequency observed in plots of membrane gain obtained using sinusoidal current injection of blowfly HS neurons by Haag & Borst (1996). Since the latter was suppressed by strong hyperpolarization of the neuron, the authors argued that this amplification is probably due to a voltage-gated regenerative conductance associated with the spikelets. In our own data, the association with spikelets is confirmed by comparing the power in the vicinity of this peak (95–105 Hz) before and after applying the spikelet filtering technique (figure 4*e*). This shows a 3.6-fold reduction in power during stimulation after spikelet removal (although the power remains significantly elevated compared with the control). The power in this region of the response spectrum is significantly higher for preferred than anti-preferred direction stimulation.

If we do a similar analysis of responses following adaptation, the converse is true: The high-frequency power following anti-preferred (hyperpolarizing) stimuli is higher than both the control and following preferred direction stimuli (figure 4*e*). While the power following preferred adaptation is substantially (*p*<0.01) decreased compared with peri-stimulus conditions, the power following anti-preferred stimulation remains elevated at a level comparable with peri-stimulus conditions. Since the increased power following anti-preferred adaptation persists even following our attempts at spikelet removal, this provides a likely explanation for the depolarizing offset seen in figure 2*d*. We thus conclude that the decrease in contrast sensitivity evident in this stimulus condition is probably the result of a fourth component of motion adaptation, not described in earlier studies—an activity-dependent, direction-selective increase in the probability of spikelet generation that boosts the overall depolarization. This leads to a direction-selective decrease in gain (for anti-preferred direction stimuli; figure 2*d*) or increase (for preferred direction stimuli; figure 2*c*) when test contrasts are close to absolute threshold.

A recent study found that local stimuli exerted a global influence on the directional gain in blowfly H1 and V1 decreasing modulation following preferred direction adaptation and increasing following anti-preferred direction adaptation (Neri & Laughlin 2005). No such effect is evident in our data (see fig. 2 in the electronic supplementary material), although in order to obtain reliable data for contrast thresholds our test stimulus was larger in angular extent than Neri & Laughlin's, making direct comparison difficult, especially as we were recording from a different neuron class and in different dipteran species.

### (f) Role of calcium

Our data support the idea that there may be four unique components of motion adaptation. While our data suggest that contrast gain and output range reductions (figure 1*c*) are most likely associated with decreased gain at the synaptic inputs to the HS cell, the after-potential and AC component are probably post-synaptic phenomena. One hypothesis for the origin of the after-potential is based on the observation that the fine distal dendrites of LPTCs that are directly depolarized by retinotopic motion input accumulate calcium during prolonged stimulation (Borst & Egelhaaf 1992; Kurtz *et al.* 2000). The intracellular calcium concentration depends on the stimulus strength, and correlates well with the after-hyperpolarization observed after motion in the preferred direction. Because calcium accumulation alone would depolarize the cells it has been suggested that the accumulated calcium opens calcium-sensitive potassium channels (Borst & Egelhaaf 1992; Kurtz *et al.* 2000). This will facilitate hyperpolarization when preferred motion ceases because sodium influx associated with the motion response no longer depolarizes the cell (Kurtz *et al.* 2000). However, a more recent study shows that elevating the cytosolic calcium concentration by ultraviolet photolysis of caged calcium does not evoke adaptation phenomena (Kurtz 2007). This makes it unlikely that adaptation in fly motion-sensitive neurons is regulated by bulk cytosolic calcium. Instead it was suggested that the direction-specific adaptation (after-hyperpolarization) is regulated by the activity of tonic sodium conductances (Kurtz 2007). Our new observation that spikelet activity is upregulated following prolonged hyperpolarization lends support to this idea, since spikelets are believed to be associated with sodium conductance (Haag & Borst 1996, 1998).

### (g) Local versus global adaptation

Nevertheless, our data confirm that contrast gain reduction observed in earlier studies (e.g. Maddess & Laughlin 1985; Harris *et al.* 2000; Kohn & Movshon 2003; Reisenman *et al.* 2003) is both locally generated and local in influence. By contrast, the MAE and our newly described AC component of adaptation both exert global influences on subsequent responses. The latter is opposite in direction to the graded MAE following preferred direction adaptation, and may reflect changes in the kinetics of the voltage-gated cation conductances believed to underlie spikelet generation (Borst & Egelhaaf 1992; Haag & Borst 1996).

Although the power spectra suggest that this AC component is more pronounced following anti-preferred direction stimuli (figure 4*e*), we also observe increased spikelet rates following preferred direction stimuli (figure 4*b*). Hence, this phenomenon is quite distinct from the classical ‘post-inhibitory rebound’ observed in many spiking neurons. The influence of this component of adaptation on subsequent responses would be greatest when the membrane potential is close to or below the resting levels, since spike amplitude is largest under these conditions (Haag & Borst 1998) and spikelets are monophasic depolarizing events in these neurons (Haag & Borst 1996). This may provide an explanation for the AC component having little effect on preferred direction stimuli, at least at higher contrasts (figure 2*c*), since strong depolarization already recruits large (and probably maximal) spikelet activity.

## Supplementary Material

Control adaptation experiments Figure 1. Control adaptation experiments

Local adaptation in hoverfly HS cells compared to blowfly H1 Figure 2. Comparison with blowfly H1 data

## References

[bib1] Barlow H.B., Hill R.M. (1963). Evidence for a physiological explanation of the waterfall phenomenon and figural after-effects. Nature.

[bib2] Beckers U., Egelhaaf M., Kurtz R. (2007). Synapses in the fly motion-vision pathway: evidence for a broad range of signal amplitudes and dynamics. J. Neurophysiol.

[bib3] Borst A., Egelhaaf M. (1987). Temporal modulation of luminance adapts time constant of fly movement detectors. Biol. Cybern.

[bib4] Borst A., Egelhaaf M. (1992). *In vivo* imaging of calcium accumulation in fly interneurons as elicited by visual motion stimulation. Proc. Natl Acad. Sci. USA.

[bib5] Borst A., Flanagin V.L., Sompolinsky H. (2005). Adaptation without parameter change: dynamic gain control in motion detection. Proc. Natl Acad. Sci. USA.

[bib6] Brenner N., Bialek W., de Ruyter van Steveninck R. (2000). Adaptive rescaling maximizes information transmission. Neuron.

[bib7] Clifford C.W., Ibbotson M.R. (2002). Fundamental mechanisms of visual motion detection: models, cells and functions. Prog. Neurobiol.

[bib8] Clifford C.W., Langley K. (1996). Psychophysics of motion adaptation parallels insect electrophysiology. Curr. Biol.

[bib9] de Ruyter van Steveninck R.R., Zaagman W.H., Mastebroek H.A.K. (1986). Adaptation of transient responses of a movement-sensitive neuron in the visual system of the blowfly *Calliphora erythrocephala*. Biol. Cybern.

[bib10] Egelhaaf M., Borst A. (1989). Transient and steady-state response properties of movement detectors. J. Opt. Soc. Am. A.

[bib11] Egelhaaf M., Kern R. (2002). Vision in flying insects. Curr. Opin. Neurobiol.

[bib12] Haag J., Borst A. (1996). Amplification of high-frequency synaptic inputs by active dendritic membrane processes. Nature.

[bib13] Haag J., Borst A. (1998). Active membrane properties and signal encoding in graded potential neurons. J. Neurosci.

[bib14] Harris R.A., O'Carroll D.C. (2002). Afterimages in fly motion vision. Vision Res.

[bib15] Harris R.A., O'Carroll D.C., Laughlin S.B. (2000). Contrast gain reduction in fly motion adaptation. Neuron.

[bib16] Hausen K. (1982). Motion sensitive interneurons in the optomotor system of the fly. II. The horizontal cells: receptive field organization and response characteristics. Biol. Cybern.

[bib17] Hausen K., Egelhaaf M., Stavenga D.G., Hardie R.C. (1989). Neural mechanisms of visual course control in insects. Facets of vision.

[bib18] Hengstenberg R. (1982). Common visual response properties of giant vertical cells in the lobula plate of the blowfly *Calliphora*. J. Comp. Physiol.

[bib19] Joesch M., Plett J., Borst A., Reiff D.F. (2008). Response properties of motion-sensitive visual interneurons in the lobula plate of *Drosophila melanogaster*. Curr. Biol.

[bib20] Kalb J., Egelhaaf M., Kurtz R. (2008). Adaptation changes directional sensitivity in a visual motion-sensitive neuron of the fly. Vision Res.

[bib21] Kohn A., Movshon J.A. (2003). Neuronal adaptation to visual motion in area MT of the macaque. Neuron.

[bib22] Kretzberg J., Warzecha A.K., Egelhaaf M. (2001). Neural coding with graded membrane potential changes and spikes. J. Comput. Neurosci.

[bib23] Kurtz R. (2007). Direction-selective adaptation in fly visual motion-sensitive neurons is generated by an intrinsic conductance-based mechanism. Neuroscience.

[bib24] Kurtz R., Durr V., Egelhaaf M. (2000). Dendritic calcium accumulation associated with direction-selective adaptation in visual motion-sensitive neurons *in vivo*. J. Neurophysiol.

[bib25] Maddess T., Laughlin S.B. (1985). Adaptation of the motion-sensitive neuron H1 is generated locally and governed by contrast frequency. Proc. Biol. Sci.

[bib26] Mather G., Verstraten F.A.J., Anstis S. (1998). The motion aftereffect: a modern perspective.

[bib27] Neri P., Laughlin S.B. (2005). Global versus local adaptation in fly motion-sensitive neurons. Proc. R. Soc. B.

[bib29] Nordström K., Barnett P.D., O'Carroll D.C. (2006). Insect detection of small targets moving in visual clutter. PLoS Biol.

[bib28] Nordström K., Barnett P.D., Moyer de Miguel I.M., Brinkworth R.S.A., O'Carroll D.C. (2008). Sexual dimorphism in the hoverfly motion vision pathway. Curr. Biol.

[bib30] Reisenman C., Haag J., Borst A. (2003). Adaptation of response transients in fly motion vision. I: experiments. Vision Res.

[bib31] Safran M.N., Flanagin V.L., Borst A., Sompolinsky H. (2007). Adaptation and information transmission in fly motion detection. J. Neurophysiol.

[bib32] Smith A.T., Scott-Samuel N.E., Singh K.D. (2000). Global motion adaptation. Vision Res.

[bib33] Solomon S.G., Peirce J.W., Dhruv N.T., Lennie P. (2004). Profound contrast adaptation early in the visual pathway. Neuron.

[bib34] Straw A.D., Warrant E.J., O'Carroll D.C. (2006). A ‘bright zone’ in male hoverfly (*Eristalis tenax*) eyes and associated faster motion detection and increased contrast sensitivity. J. Exp. Biol.

[bib35] Warzecha A.-K., Egelhaaf M., Zanker J.M., Zeil J. (2001). Neural encoding of visual motion in real-time. Motion vision.

