# Supplementary material for: The motion after-effect: local and global contributions to contrast sensitivity
Source: Proc Biol Sci. 2009 Feb 25;276(1662):1545–54. doi: 10.1098/rspb.2008.1932 (PMC2660997; doi:10.1098/rspb.2008.1932)
Supplement: Control adaptation experiments — Figure 1. Control adaptation experiments [file rspb20081932s01.pdf]

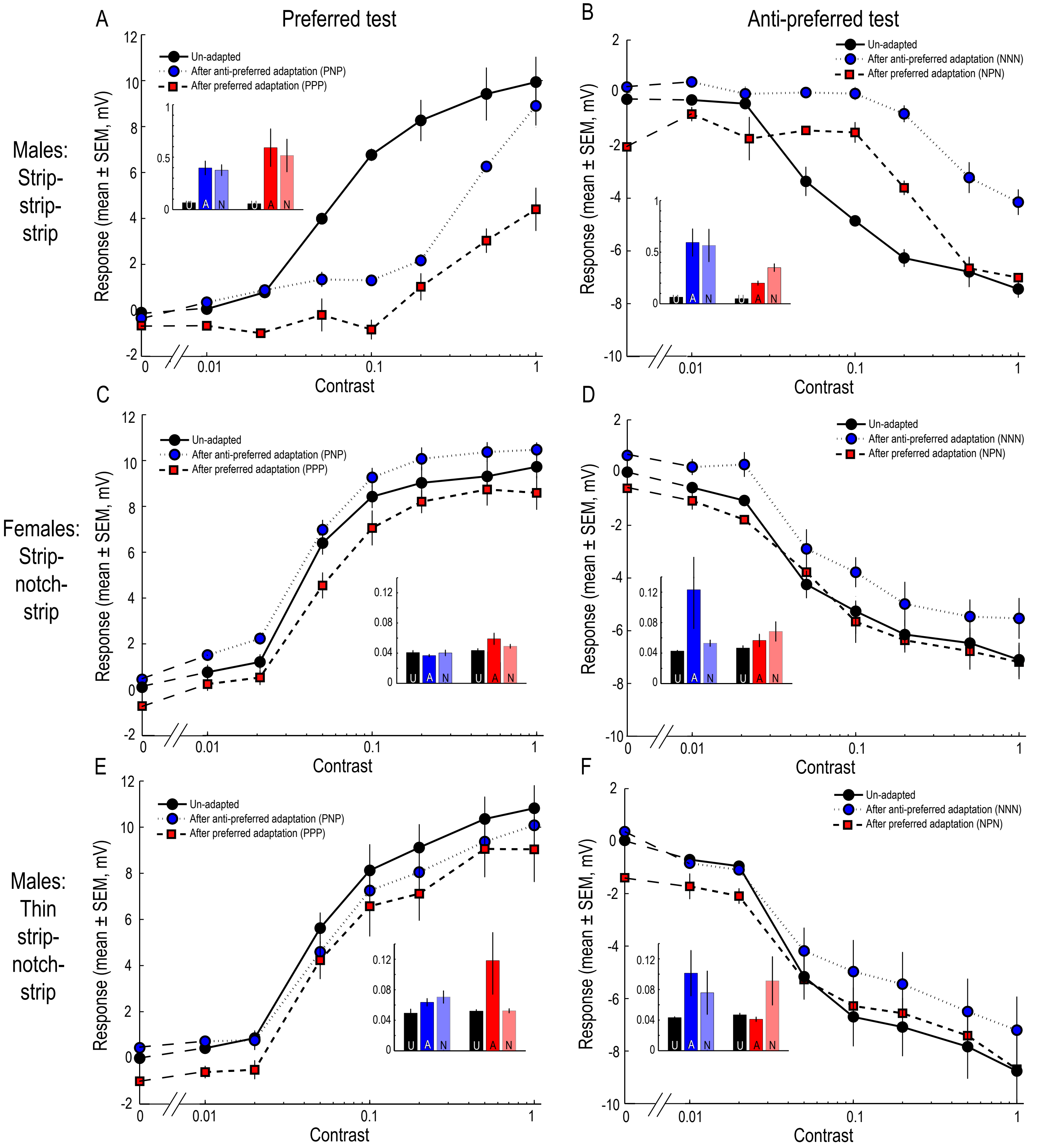

Control adaptation experiments

A) *Same-location adaptation causes a large contrast gain reduction.* The graph shows the unadapted (black circles) and adapted contrast response function to preferred direction motion (0.1 cpd, 5 Hz) following adaptation (0.1 cpd, 20 Hz) either in the anti-preferred (PNP, blue circles) or preferred (PPP, red squares) direction. The local 'strip' grating was used for both test and adaptation. The inset shows C_{50} measured in the unadapted, adapted and normalized (adapted response – antagonistic after-potential) data. B) The unadapted and adapted contrast response function to anti-preferred direction motion with adaptation either in the anti-preferred (NNN, blue circles) or preferred (NPN, red squares) direction. Data displayed as mean ± SEM, n = 4.

C) *Local adaptation in female hoverflies.* The unadapted and adapted contrast response function to preferred direction motion (0.1 cpd, 5 Hz) following adaptation (0.1 cpd, 20 Hz) either in the anti-preferred or preferred direction in female hoverflies. The inset shows C_{50} measured in the unadapted, adapted (red and blue) and normalized (shaded red and blue) data. D) The unadapted and adapted contrast response function to anti-preferred direction motion with adaptation either in the anti-preferred or preferred direction. All data displayed as mean ± SEM, n = 6.

E) *Local adaptation with a thinner strip and a wider gap to the adapting pattern.* The graph shows the unadapted and adapted contrast response function to preferred direction motion with adaptation either in the anti-preferred or preferred direction. We used a thinner strip sine-wave grating as test and a wider gap to the notch adapting pattern. The inset shows C_{50}. F) The unadapted and adapted contrast response function to anti-preferred direction motion with adaptation either in the anti-preferred or preferred direction. All data displayed as mean ± SEM, n = 4.
