# Supplementary material for: The motion after-effect: local and global contributions to contrast sensitivity
Source: Proc Biol Sci. 2009 Feb 25;276(1662):1545–54. doi: 10.1098/rspb.2008.1932 (PMC2660997; doi:10.1098/rspb.2008.1932)
Supplement: Local adaptation in hoverfly HS cells compared to blowfly H1 — Figure 2. Comparison with blowfly H1 data [file rspb20081932s02.pdf]

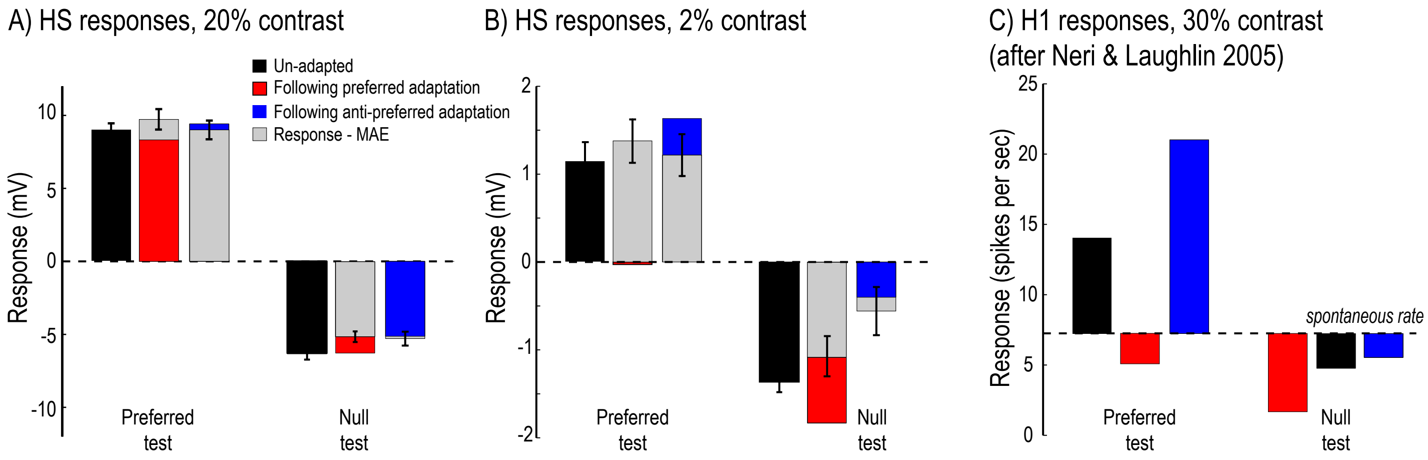

Local adaptation in hoverfly HS cells compared to blowfly H1

A) The graph on shows the un-adapted (black) responses to preferred (left) and anti-preferred (right) direction motion at a contrast of 20%. The adapted responses to the same contrasts are shown in red (following preferred direction adaptation) and blue (following anti-preferred direction adaptation). The grey bars show the same responses after subtracting the antagonistic after-potential measured at a test contrast of 0 (see Figure 2). The data (n = 15) is reproduced from Figure 2. B) The graph shows the un-adapted (black) responses to preferred (left) and anti-preferred (right) direction motion at a contrast of 2%. The adapted responses to the same contrasts are shown in red (following preferred direction adaptation) and blue (following anti-preferred direction adaptation). The grey bars show the same responses after subtracting the antagonistic after-potential measured at a test contrast of 0 (see Figure 2). The data (n = 15) is reproduced from Figure 2. C) The graph shows results from Neri & Laughlin (2005) with H1 un-adapted (black) responses to preferred (left) and anti-preferred (right) direction motion at a contrast of 30%. The adapted responses to the same directions are shown in red (following preferred direction adaptation) and blue (following anti-preferred direction adaptation). The data is reproduced from Figure 1E in Neri & Laughlin, 2005.
